# Research Progress of Fever with Thrombocytopenia Syndrome

**DOI:** 10.1007/s44231-023-00035-6

**Published:** 2023-03-23

**Authors:** Ning Luo, Mengdie Li, Ming Xu, Chuanchuan Shi, Xinge Shi, Rong Ni, Yu Chen, Liang Zheng, Yuling Tu, Dan Hu, Chunlin Yu, Qingying Li, Yibin Lu

**Affiliations:** General ICU, Xinyang Central Hospital, Xinyang Key Laboratory of Critical Care Medicine, Xinyang, 464000 Henan China

**Keywords:** The novel bunyavirus, Epidemiology, Route of Transmission, Pathogenesis, Clinical manifestation, Treatment

## Abstract

Severe fever with thrombocytopenia syndrome (SFTS) is a new infectious disease first discovered in Ta-pieh Mountains in central China in 2009. It is caused by a novel bunyavirus infection (SFTSV). Since the first discovery of SFTSV, there have been case reports and epidemiological studies on SFTS in several East Asian countries, such as South Korea, Japan, Vietnam and so on. With the rising incidence of SFTS and the rapid spread of the novel bunyavirus around the world, it is clear that the virus has a pandemic potential and may pose a threat to global public health in the future. Early studies have suggested that ticks are an important medium for the transmission of SFTSV to humans; in recent years, it has been reported that there is also human-to-human transmission. In endemic areas, potential hosts include a variety of livestock and wildlife. When people are infected with SFTV, the main clinical manifestations are high fever, thrombocytopenia, leukocytopenia, gastrointestinal symptoms, liver and kidney function damage, and even MODS, with a mortality rate of about 10–30%. This article reviews the latest progress of novel bunyavirus, including virus transmission vector, virus genotypic diversity and epidemiology, pathogenesis, clinical manifestation and treatment.

## Introduction

Severe fever with thrombocytopenia syndrome(SFTS) is an infectious disease caused by viral infection. It was first reported in 2009 in the Ta-pieh Mountains in central China, and then in 2010, Yu et al. [1]. isolated this pathogen as a virus, known as severe fever with thrombocytopenia syndrome virus (SFTSV), which belongs to the Bunyaviridae, also known as the novel bunyavirus. The International Committee on Taxonomy of Viruses named the virus Dabie bandavirus in 2019, which belongins to Bandavirus genus,Phenuiviridae family, Bunyavirales order. But SFTSV is currently the most widely used in the world [2]. The appearance of SFTSV is spherical, with a diameter of 80–100 nm. It consists of a single strand of negative strand RNA, including three fragments: large (L), medium (M) and small (S). L-segment encodes RNA-dependent RNA polymerase (RdRp), which functions as a transcriptase / replicase of the virus [3]. The M segment encodes the viral surface glycoproteins, which consists of two glycoproteins, Gn and Gc. They form the envelope of the virus. This envelope has antigenic characteristics, after the virus invades the body, it can stimulate the body to produce specific neutralizing antibody recognition targets, these characteristics can provide a direction for future vaccine research. Therefore, the study of viral envelope Gn/Gc and the analysis of antigenic characteristics are very important. S-segment is a double RNA, which is composed of coding nuclear protein Np and non-structural protein NSs. Np plays an important role in viral RNA encapsulation/RNP complex, and NSs interferes with host interferon production [4].Currently, there are no effective therapeutics or vaccines to combat the infection of SFTSV. Hence, it is imperative for the further study of SFTSV. This review mainly focuses the studies of SFTS disease in epidemiology, transmission, pathogenesis and countermeasures.

## Vector and Disease Transmission

The life cycle and mechanism of continuous transmission of SFTSV in nature are not clear [5]. Through the existing research confirmed that SFTSV is mainly transmitted by ticks, and most of the patients initially diagnosed as SFTS have a history of tick bites before the onset of SFTS [6]. Ticks are not only the main transmission vector of SFTSV, but also the important storag through eggs. Haemaphysalis longicornis are mainly distributed in the Ta-pieh Mountains in central China, including Henan, Hubei and Anhui provinces.SFTS occurrence probability is high when altitude is between − 100 m and 100 m, and the probability is nearly 0 when altitude is beyond 3000 m [7]. Haemaphysalis longicornis has a wide range of hosts, including wild and domestic mammals and birds, and is unique to the Asia–Pacific region in terms of distribution [8].Asian longhorned ticks, probably transported by migratory birds, play a major role in the rapid spread of SFTSV [9].

SFTS RNA can be detected in arthropods of ticks such as Haemaphysalis, Ixodes minimus, Ixodes tortoise, Ixodes asiatica and Ixodes japonicus in the endemic areas of SFTSV. Another feature of these tick species is that they can reproduce parthenogenetically and survive in a variety of environmental conditions [10]. Although these tick species carry a novel bunyavirus, further studies are needed to confirm whether the virus can be transmitted to animals or humans [8]. Because the ability of SFTSV transmission and infection depends on the ability of tick species to enlarge and transmit the virus to animals and humans [11]. Recently, Haemaphysalis longicornis has been found in the United States and is spreading rapidly. It has been reported that someone has been bitten by Haemaphysalis longicornis [12]. The Department of Disease Control and Prevention (CDC) of the United States has designated SFTA as one of the top ten infectious diseases and has conducted surveillance studies on ticks. Fortunately, no RNA fragment of the novel bunyavirus was found in Haemaphysalis longicornis[12].

**Fig. 1:**
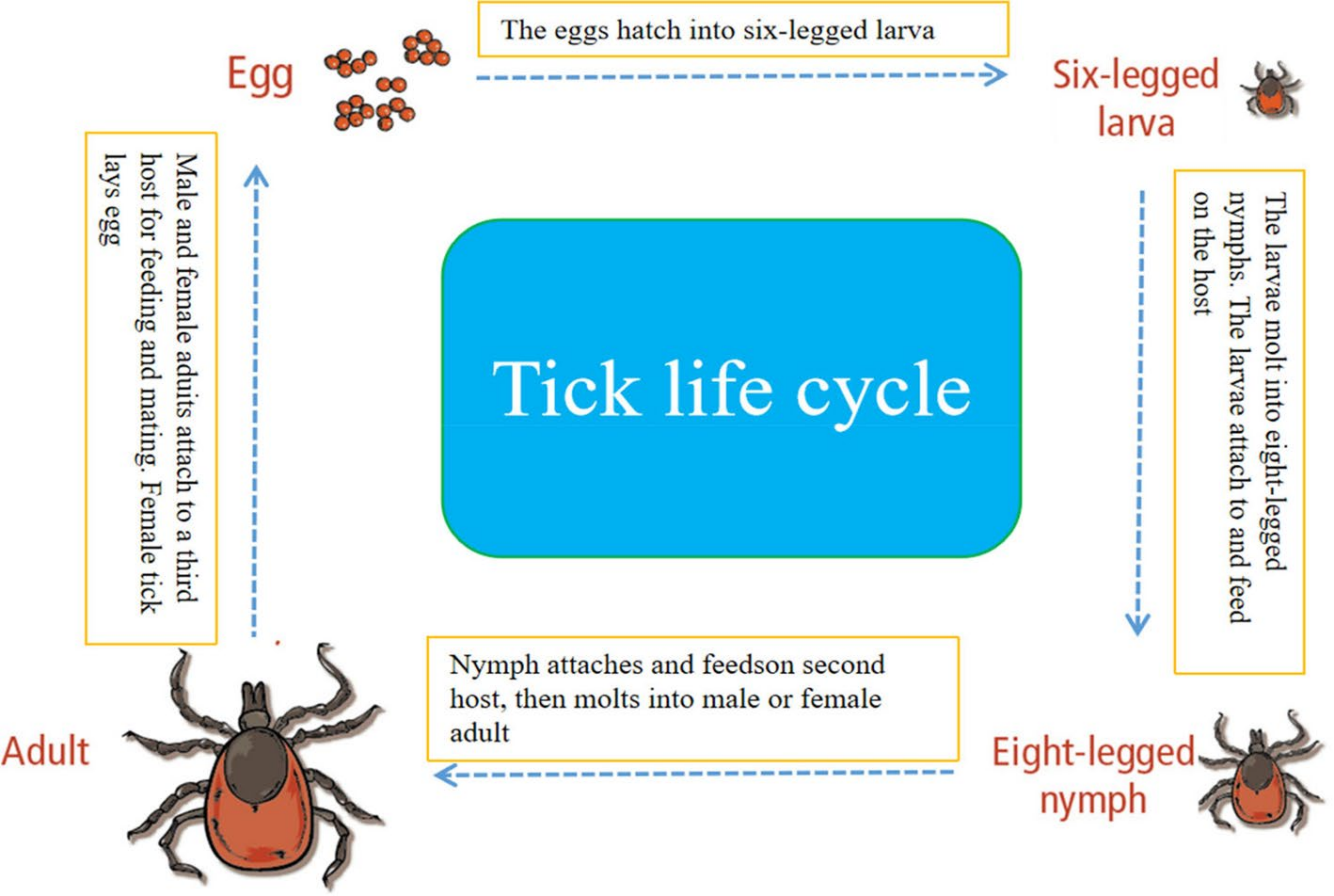
Tick life cycle

SFTSV maintains virus replication in nature through tick-to-tick cycles (through egg or cross-worm, the virus spreads from adult ticks to young ticks, or by co-feeding on the same host). In the tick cycle, ticks act not only as a carrier, but also as a host of SFTSV [13] (Fig. 1).

SFTSV can also be detected in livestock and wild animals. Most animals are found to be subclinical infected with SFTSV. The reason for subclinical infection is that infected animals do not have prolonged symptoms of viremia and viral infection. SFTSV's RNA can be detected in cattle, goats, pigs, chickens, dogs, cats, deer, hedgehogs and minks, but these animals did not develop the disease. The novel bunyavirus is transmitted to these vertebrates through tick bites, which serve as storage hosts for SFTSV. This cycle makes SFTSV spread in nature to achieve a lasting cycle. Human beings usually have a lot of contact with domestic animals and need special precautions. There is also a potential risk of cross-regional transmission of migratory birds infected with SFTSV [14]. Different factors affect the infection rate coefficients of different transmission routes. Sunshine duration, relative humidity, temperature and tick density are important factors affecting the occurrence of SFTS. Hurricanes reduce the incidence of SFTS in the short term, but have little effect in the long term. The most effective intervention to reduce the incidence of SFTS is to reduce population exposure to high-risk environments [15].

Tick bites are the main route of transmission of SFTSV, as initial epidemiological reports show that patients diagnosed with SFTS have a history of tick bites before the onset of SFTS [16]. With the increase of SFTS cases and the improvement of epidemiological investigation, it was found that human-to-human transmission of SFTSV infection occurred [17]. Recently, it has been reported many times that medical staff and family members have direct blood contact with patients with SFTS without wearing protective equipment, which leads to aggregation infection [18, 19]. Laboratory tests showed that after SFTSV infection, the viral load of serum, sputum, saliva, cerebrospinal fluid, semen and urine were higher [20]. All data above shows that SFTSV could be transmitted from person to person by direct contact of body fluid. Especially, the health care worker should take preventive measures to prevent infection.

## Prevalence of SFTSV in Vertebrates

Haemaphysalis longicornis of Asia undergo three unique life stages (larva, nymph and adult). At each stage, they are parasitic on a variety of wild and domestic animals, including birds, livestock, etc.There is a close relationship between humans and animals, especially in SFTSV-endemic areas, which increases the risk of SFTS epidemic [21]. Ticks spread the virus to wild or domestic animals after biting them. These animals may have no clinical symptoms, but their secretions contain SFTSV RNA, which can be transmitted to humans after contact [22].SFTSV antibodies can be detected in many animals, including goats, sheep, cattle, dogs, pigs, chickens, cats, rodents, deer, wild boars and hedgehogs[23]. A cross-sectional cohort study conducted by virologists in China also revealed the infection rate of livestock, with a relatively high serum positive rate of 69.5% in sheep, 60.4% in cattle, 37.9% in dogs and 47.4% in chickens, while the prevalence rate in pigs was only 3.1% [24]. Differences in infection rates among animals may depend on the number of ticks parasitized on them. Sheep, goats and cattle are usually kept free, which makes them vulnerable to tick bites in nature.And it makes them more likely to get SFTSV infection. Interestingly, although rodents are also known hosts of Bunya virus, the serum positive rate of SFTSV in rodents is much lower than that in domestic animals [25]. These susceptible vertebrate hosts are necessary to establish and maintain arbovirus transmission cycles. It is not clear whether long-term or persistent infection will occur in animals infected with SFTSV.

## Geographical Distribution and Genetic Diversity

SFTS was first reported in the Ta-pieh Mountains in central China in 2009, and quickly spread to Zhejiang, Jiangsu, Shandong and other regions. Japan and South Korea also reported cases of SFTS in 2012 [26, 27]. Recently, Vietnam and Taiwan have also reported SFTS-related cases [28, 29].

The mechanism of rapid spread of severe fever with thrombocytopenia syndrome is unclear. However, the spread of the virus is usually attributed to two main mechanisms:increased contact between wildlife and human populations and geographical spread of hematophagous arthropod vector or their vertebrate host outside the area of endemicity. Haemaphysalis longicornis is also a common parasite of migratory birds, which usually breeds and migrates between the endemic areas of China, Korea and Japan, and the endemic areas of STFS match the migration routes of migratory birds [30]. However, further field studies are needed to understand the relationship between tick vector habitats and tick infection rates in migratory birds in China, Korea and Japan. Understanding the transmission mechanism is very important for in-depth understanding of the ecological epidemiology of the virus within its epidemic range.

Several studies have highlighted the genetic diversity of SFTSV. The novel bunyavirus consists of several gene fragments. Because it is a RNA virus, the gene mutation caused by lack of proofreading function of SFTSV in viral RNA replication and transcription, which will lead to gene recombination. As a result, genetic diversity and new genome lines have emerged in different hosts [31]. According to the suggestion of Professor Fu of Fudan University in Shanghai, the phylogenetic analysis of SFTSV strain can be divided into 6 genotypes, which are called genotypes A to F [32]. The prevalence of SFTSV genotypes is also different in different countries. Recently, Yun et al. have proved in the laboratory that genotype B can be further subdivided into three different gene subtypes, Bmuri 1, Bmai 2 and Bmi 3 [33]. There are at least 9 different recombinant genes in Korea and 7 SFTSV recombinant genes reported in China, which indicates that the SFTSV virus strain is undergoing continuous evolution in nature [24, 25]. Reported SFTS case fatality rates vary widely in affected countries in East Asia, which is estimated to be related to different genotypes of human infection. At present, it is reported that the high mortality rates in Japan and South Korea are 27% and 23.3% respectively. By contrast, the mortality rate of SFTSV in China is about 10–30% [24]. Some virologists further subdivided the most popular genotypes in Japan and Korea as Bmur2, with an incidence of 86% and 36.1% respectively [34]. In China, the most common incidence of genotype F is 43.6%, followed by the incidence of genotype An at 20.1% [24]. Therefore, this suggests that the reported difference in mortality may be related to the different distribution of SFTSV genotypes, and Yun et al. in Korea further support this view [25]. The above studies on genotypes suggest that the morbidity and mortality in epidemic areas are related to the genotype of the virus, but it should also be noted that age, sex and underlying diseases are also closely related to mortality.

## Clinical Manifestations of Patients with SFTS

The main clinical symptoms of SFTSV infection are fever and thrombocytopenia. In addition to these two major clinical manifestations, it also includes gastrointestinal symptoms such as poor anorexia of nausea and vomiting, leukocytopenia, low lymphocyte count, coagulation dysfunction and hemorrhagic tendency. In addition, there are also reported cases of atypical and asymptomatic SFTS infection [6]. The incubation period of SFTS is generally 7–14 days (average 9 days), and it’s clinical course can be divided into three different stages according to the progression of the disease: fever stage, multiple- organ dysfunction (MOD) stage and convalescent stage. In the stage of fever, the patient initially presented with acute high fever and high serum viral load, and the highest body temperature was 39–40 ℃, accompanied by thrombocytopenia, leukocytopenia and lymphadenopathy. Laboratory examination showed that the circulating threshold of real-time quantitative PCR of novel bunyavirus RNA was very low, and the viral load was very high [32]. It usually takes 5–7 days for the disease to develop into MODS from the febrile phase. The characteristics of MODS phase are as follows: Hematological symptoms include hemorrhagic manifestations, persistent decrease in platelet count, persistent abnormality of blood coagulation, and disseminated intravascular coagulation (DIC);Neurological symptoms include drowsiness, muscle tremors, convulsions, convulsions and coma; Respiratory symptoms include shortness of breath and decreased blood oxygen saturation. The circulatory system will have shock manifestations such as decreased blood pressure, increased heart rate, and systemic hypoperfusion. Of course, there will be jaundice, anuria and other manifestations of liver and kidney failure. These manifestations occur at the end of the disease, and are often sequential organ function damage, developing into severe, critical, which is also the direct cause of death of patients [35]. Some patients with mild symptoms can progress directly from the febrile period to the recovery stage. Other clinical aspects of SFTS included the increase of activated partial thromboplastin time (APTT), prothrombin time (PT), serum alanine aminotransferase (ALT), aspartate aminotransferase (AST), blood urea nitrogen (BUN), creatinine (Cr), lactate dehydrogenase (LDH) and myocardial enzymes [36].

As early as 2013, according to the clinical characteristics of Chinese patients, clinical experts divided the severity of SFTS into mild, severe and critical. After classification, it is beneficial to focus on treatment and find critical patients in time. The patients with T < 38 ℃, muscle soreness, digestive system symptoms such as anorexia, diarrhea, nausea and vomiting, no systemic bleeding tendency, clear mind and no nervous system symptoms were divided into mild symptoms. The patients with T > 38 ℃, obvious symptoms of general fatigue, severe anorexia; mental malaise, accompanied by neurological symptoms, such as lethargy, muscle tremor, systemic involuntary tremor, convulsions, convulsions and delirium are divided into severe cases.On the basis of severe cases, patients with any of the following conditions such as: ARDS, heart failure, AKI, sepsis or septic shock; DIC, encephalitis, that is, summarized as critical [37]. The above clinical classification is very important for front-line doctors. The doctors can identify severe and critical patients in time, and include patients in ICU, so as to strengthen the monitoring of patients and support treatment for their organs.

## Pathogenesis of SFTSV

**Fig. 2:**
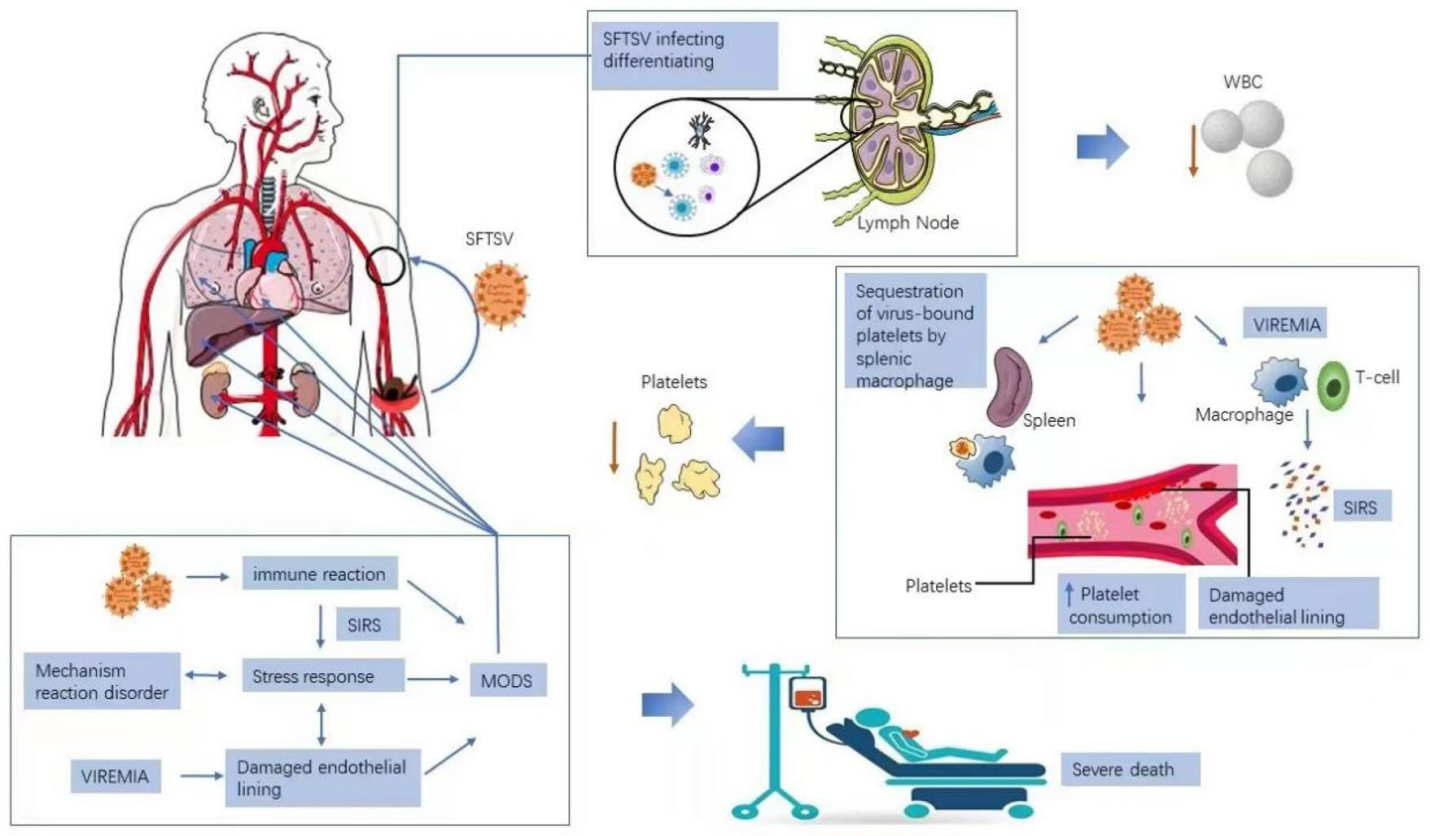
The mechanism of SFTSV pathogenesis. SFTSV transmission to humans commonly occurs from virus-carrying-tick-bite. The SFTSV then invades the lymph node nearest to the tick-bite wound, targeting immune cells such as B-cells, impairing host immune response from invading pathogen.It also leads to a decrease in white blood cells.After further replication, the virus goes to the systemic circulation, in response to viremia, other immune cells are over-stimulated causing cytokine storm and severe inflflammatory response syndrome(SIRS). SIRS causes damage to the vascular endothelium, which allows platelets to adhere and aggregate.Thrombocytopenia is a hallmark of SFTSV infection.Body response disorder and stress response caused by cytokine storm lead to multi-organ dysfunction, reflflected by the elevation of liver, kidney, and heart serum markers. Severe cases often die from MODS

Pathological study is of great significance for understanding the pathogenesis of the virus. Autopsy of patients who died of SFTS can better understand it’s pathogenesis. A pathological study of SFTS suggests that biopsy of lymph nodes near tick bites indicates necrotizing lymphadenitis, which is consistent with the signs of SFTS patients. These patients are prone to enlarged lymph nodes at the bitten site [38]. In addition to the involvement of the surrounding lymphatic system, another important pathological feature of SFTS is that non-lymphoid organs such as solid organs (lungs, liver, kidney, etc.) will also be involved, which is consistent with multiple organ failure in critically ill patients. Some studies suggest that SFTSV is not detected in parenchymal cells, but SFTSV is found in B lymphocytes in capillaries of non-lymphoid organs. B lymphocytes are the center of humoral immune response and are responsible for mediating the production of antigen-specific immunoglobulins [39]. Immunoglobulin (IgG) is essential for neutralizing and eliminating viruses. However, it was found that the titer of IgG antibody produced by B lymphocytes in patients with severe SFTS was very low. This situation may suggest that the humoral immunity mediated by B lymphocytes is damaged when the novel bunyavirus is infected, especially in severe patients (Fig. 2). In addition, the analysis of severe SFTS cases showed that most of the peripheral plasma mother cells did not express IgM and IgG, which suggested the deficiency of humoral lymphocyte immune response. Moreover, neutralizing antibody IgG could not be detected in severe SFTS cases, which may be due to the inability of IgG-positive B cells to perform class conversion at the time of infection [40]. Taken together, these findings suggest that in severe SFTSV infection, the virus interferes with the differentiation of B lymphocytes in secondary lymphoid organs. The susceptibility of SFTSV to B lymphocytes and how the virus destroys the humoral immune mechanism of the body will be an important topic worth exploring.

The immunopathogenesis of SFTSV infection is complex, which includes a cascade of reactions involving a wide range of immune cells, inflammatory mediators, inflammasomes and signaling pathways [41]. SFTSV affects the differentiation of B lymphocytes and leads to humoral immunity deficiency. Inflammatory storm of cytokines caused by viral infection is also considered to be a major pathological feature of patients with s evere SFTS. Studies on the expression of cytokines and chemokines in patients with SFTS have shown that levels of IL-6, IL-10, IFN-γ-inducible protein (IP)-10 and IFN- γ usually increase in the early stages of the disease [42]. Professor Kwon's research says there is a significant correlation between IP-10 levels and viral load.But because of the small sample size, further research is needed to confirm this [43]. In addition, the relationship between cytokine / chemokine levels and platelet count and serum enzymology (AST, LDH, ALT) has also been reported [38, 39]. The decrease of platelet level is related to the decrease of soluble CD40 ligand and platelet-derived growth factor BB. Similarly, when IL10, IL-2 receptor and IP-10 levels increased, platelet levels decreased [39]. The serum levels of AST, ALT and LDH were positively correlated with IL-10, sIL-2RA, HSP70, IP-10, IL-4, IFN- γ, tPAI-1 and other inflammatory / chemokines. In addition, it is reported that IL-10 is oversecreted in severe SFTS, which can induce compensatory anti-inflammatory response syndrome and trigger abnormal and uncontrolled inflammatory disorders, thus causing damage to various organs of the body [44].

## Treatment of SFTS

### Ribavirin

At present, the main treatment for SFTS disease is symptomatic treatment and traditional Chinese medicine treatment. Symptomatic treatment is to deal with fever, give drugs to increase platelet and leukocyte use.For example, the Chinese medicine Xuebijing injection can reduce SIRS reaction. It can also reduce the DIC score of SFTS and restore the platelets to normal [41]. According to the national guidelines for the prevention and control of SFTS issued by the Ministry of Health of China in 2010, ribavirin, a synthetic nucleoside antiviral drug, is recommended for clinical treatment of SFTS [47]. Ribavirin is a synthetic nucleotide antiviral drug that exhibits broad-spectrum antiviral activity against a variety of viruses, such as respiratory syncytial virus, influenza, measles, herpesvirus, and in combination with interferon (IFN)-α can inhibit hepatitis C. A study by Professor Lee from Korea showed that ribavirin inhibited SFTSV replication in SFTS patients after 48 h of ribavirin use [45]. Although the successful treatment of ribavirin has been reported, the use of ribavirin did not reduce the final outcome of SFTS in more than one study. Professor Liu Wei of the Beijing Institute of Microbiology conducted a retrospective study on the 154 Hospital of the people's Liberation Army in Xinyang City, Henan Province. The professor selected a total of 311 cases, including 54 deaths. They found that there was no increase in platelet count and no decrease in viral load in patients treated with ribavirin compared with patients who did not receive ribavirin. He even found that patients who received ribavirin had lower platelet counts than those who did not receive treatment [46]. The therapeutic effect of ribavirin on SFTSV infection often depends on the viral load, which is less than 1 copy, and the therapeutic effect of ribavirin is better in the early stage of the disease [47]. Although there are many studies on SFTSV, the pathogenesis of the virus is not clear. The number of antiviral drugs against SFTSV is limited. More importantly, obtaining new drugs through the basic research process is a very long-term process. However, it is a relatively fast method to evaluate the efficacy of existing antiviral drugs.

### Favipiravir

Favipiravir, discovered and synthesized by Toyama Chemical Co., Ltd., Japan, is a new antiviral drug so far. It has a wide range of activities against a variety of RNA viruses, including influenza virus, Bunya virus, West Nile virus, yellow fever virus and foot-and-mouth disease virus [48]. It’s mechanism is that Favipiravir ribosyl-5-triphosphate inositol (fapiravir RTP) is produced by phosphoribosylation by host cell enzymes, and the viral RNA polymerase mistakenly recognizes Favipiravir RTP, which inserts Favipiravir RTP into the viral RNA chain or binds to the viral RNA polymerase domain, thus hindering the replication and transcription of the viral RNA chain [49].

In many animal models of SFTSV infection, the efficacy of Favipiravir in vivo has been fully verified. One study showed that when treatment began 3 days or earlier after SFTSV infection, all mice treated with Favipiravir survived, even though mice treated 4 and 5 days after infection showed a survival rate of 83% and 50%, respectively [49]. At present, there are few reports on the clinical use of Favipiravir in the treatment of severe fever with thrombocytopenia syndrome, which needs more attention.

### Calcium Channel Blockers

Calcium channel blockers(CCBs) can reduce intracellular Ca_2_^+^ levels and are widely used in the treatment of various cardiovascular diseases, including hypertension, angina pectoris and supraventricular arrhythmias. In recent years, some studies have shown that CCBs has strong antiviral activity against Ebola virus, Marburg virus and Japanese encephalitis virus [50, 51]. Among many calcium antagonists, two drugs, benidipine hydrochloride and nifedipine, can inhibit Ca_2_^+^ replication by reducing virus-induced SFTSV influx [52]. Professor Li's study further analyzed the anti-sftsv effects of these two kinds of CCB in C57BL/6 mice and humanized mice [49]. It was found that the two drugs could reduce viral load, increase platelet count and reduce mortality in humanized mouse model. They also conducted a retrospective clinical study of SFTS patients, including 83 patients who received nifedipine (nifedipine before and during hospitalization), 48 patients who did not receive nifedipine (nifedipine before admission but not nifedipine during hospitalization), and 249 general SFTS patients who did not take nifedipine at all.It was found that the case fatality rate of nifedipine treatment group (3.6%) was 5 times lower than that of general SFTS treatment group (19.7%) or non-nifedipine treatment group (20.8%). The results of these studies show that CCBs has potential therapeutic effects on patients with SFTS [53]. In the future, we can consider a prospective way to evaluate the antiviral efficacy of CCBs in SFTS.

### Immunoglobulin (IVIG)

IVIG plays an important role in the treatment of various viral diseases by triggering complement activation, virus neutralization, antibody-dependent cytotoxicity and conditioning. Based on the mechanism of immunosuppression and inflammatory factor disorder in the pathogenesis of SFTS, we believe that IVIG can help reduce viral load in patients with SFTS, inhibit SFTSV transmission, and effectively inhibit cytokine storm. However, there are only a few cases of successful use of IVIG in patients with severe SFTS [54]. The mechanism of IVIG in patients with SFTS is not completely clear, which needs to be further confirmed by clinical data.

### Glucocorticoid

Severe SFTS can lead to rapid deterioration of the disease, and patients are easy to die of septic shock and MODS,DIC. The key to this pathogenic process is the cytokine storm. Considering the pathogenesis of SFTS, clinicians believe that steroids are a therapeutic option for suppressing the immune system of patients with severe SFTS. Kim et al. [54] reported two cases of successful treatment of SFTS with glucocorticoid combined with intravenous immunoglobulin (IVIG) in South Korea. Nakamura et al. [55] reported that steroids were effective in 3 Japanese patients with SFTS complicated with encephalopathy. Glucocorticoid therapy may increase the chance of secondary infection, especially fungal infection. Because SFTS patients themselves may have secondary fungal infection, and there is evidence that SFTS patients with secondary fungal infection are more ill and have a higher mortality. Based on this situation, we do not recommend glucocorticoid as a routine treatment for SFTS, especially in patients with mild or mild symptoms within 5 days after the onset of symptoms [56].

### Plasma Exchange (TPE)

SFTSV infection can lead to a storm of cytokines all over the body, which can damage organ function. Plasma exchange may be a possible rescue method for the removal of cytokines. Korean scholars have done much research on this therapy. A Korean hospital reported 2 cases of SFTS treated with therapeutic Plasma exchange(TPE) and oral ribavirin [57]. Another study on the use of TPE in SFTS patients found that the clinical and laboratory parameters of most SFTS patients improved rapidly after plasmapheresis [58]. Statistical analysis of some studies on TPE treatment of SFTS shows that there is no significant difference between the TPE group and the non-TPE group. In addition, TPE also has side effects [59]. The above TPE studies are limited because they show only a relatively small number of clinical samples and there is no strict comparison. Doctors still need to observe the clinical manifestations of patients and use TPE cautiously.

### Vaccine Research of SFTSV

Vaccine is the most effective way to deal with infectious diseases, and the development of a vaccine against the novel bunyavirus can provide mass immunity to people living in epidemic areas. In 2019, researchers at the University of the Chinese Academy of Sciences published a paper on a novel bunyavirus vaccine using recombinant vesicular stomatitis virus (rVSV) as a vector. In this study, VSV has been developed as a promising attenuated virus vaccine vector to induce the production of neutralizing antibodies that can effectively resist the deadly challenges of a variety of pathogens, including Hendra virus, Lassa virus, Ebola virus, Marburg virus, Nipah virus, etc. [60].

VSV is classified as a RNA virus of the family Rhabdoviridae, which is covered with an envelope protein when it sprouts. The envelope protein is composed of two substances, one is lipid bilayer and the other is anchoring protein derived from glycoprotein. The reason why VSV is used as a vaccine vector and model is that it’s envelope protein can be recombined with foreign virus envelope protein to form virus particles, and the antigen composition remains unchanged. The effects of recombinant vesicular stomatitis virus in invading the body, being recognized by the immune cells of the body, binding to the receptor, and producing neutralizing specific antibodies are basically similar to that produced by SFTSV invading the body [61, 62]. Therefore, using rVSV to develop SFTSV vaccine will have a great prospect.

### Treatment of MODS

SFTS is prone to sepsis and MODS [37]. In principle, all patients with MODS should be admitted to ICU for emergency treatment. At present, the main treatment includes etiology treatment and organ function support.

The cause of MODS was actively eliminated. For SFTS patients, the cause was the storm of cellular inflammatory factors caused by viral infection. If septic shock is present, tissue and organ perfusion should be improved as soon as possible to avoid further aggravating organ function damage. If a bacterial infection presents clinically, combined antibiotic therapy is required [42].

The main measures to improve oxygen metabolism and correct tissue hypoxia include increasing oxygen supply, reducing oxygen consumption and improving the ability of tissue cells to use oxygen. At present, increasing oxygen supply is the most feasible means to improve tissue hypoxia, which requires three conditions: ① normal hemoglobin content; ② Oxygen therapy, if necessary, mechanical ventilation to support breathing, so that SaO2&gt; 90%; ③ Normal cardiac function and effective circulating blood volume. Vasoactive drugs can be used appropriately to maintain tissue and organ perfusion. Reducing oxygen consumption is often overlooked and can be achieved through sedation, hypothermia and mechanical ventilation to support breathing.Respiratory support treatment Respiratory support is one of the important means to improve oxygen delivery and reduce oxygen consumption. When selecting ventilator mode and setting ventilator parameters, attention should be paid to the prevention and treatment of ventilator related lung injury, and the influence of mechanical ventilation on organ function should be reduced as far as possible [63].

## General Conclusions

SFTSV has the potential of pandemic, has an impact on global public health and poses a great threat. People in affected areas are at risk of being bitten by ticks when engaged in agricultural and outdoor activities. With the migration of migratory birds, virus spillovers often spread between humans, animals and ticks. Some studies have investigated the complex interaction between SFTSV and the host immune system. For example, the failure to produce a virus-specific humoral response is attributed to b-cell dysfunction. It has also been reported that B cell differentiation in secondary lymphoid organs is the main target of the virus at the end of fatal SFTSV infection. In addition, an inflammatory storm caused by an overimmune response to the virus can lead to progressive sexual organ damage and death. The mainly reasons are: First, the early pathogenesis of SFTSV infection; Second, the similarities and differences and interrelationship between the pathogenesis of fatal and surviving SFTSV infection. At present, there is no specific antiviral drug against SFTSV infection, nor is there a commercial vaccine. Redeveloping new antivirals is a promising approach, but it consumes time and resources. Therefore, more and more people are looking for candidate treatments for emerging viruses through the reuse of existing antiviral drugs. Vaccines are the most powerful tool to prevent outbreaks in response to this infectious disease, and some vaccine-related studies have been published. As the research on SFTSV is still in its infancy, there are still many problems to be solved.

## Data Availability

The datasetsused and/or analyzed in the present study are available from the corresponding author on reasonable request.
